# Imepitoin Shows Benzodiazepine-Like Effects in Models of Anxiety

**DOI:** 10.3389/fphar.2018.01225

**Published:** 2018-11-01

**Authors:** Odilo Engel, Aleksandar Masic, Gary Landsberg, Melissa Brooks, Daniel S. Mills, Chris Rundfeldt

**Affiliations:** ^1^Boehringer Ingelheim Vetmedica GmbH, Ingelheim am Rhein, Germany; ^2^CanCog Technologies Inc., Toronto, ON, Canada; ^3^Animal Behaviour, Cognition and Welfare Group, School of Life Sciences, University of Lincoln, Lincoln, United Kingdom; ^4^Drug Consulting Network, Coswig, Germany; ^5^Department of Pharmacology, Toxicology and Pharmacy, University of Veterinary Medicine Hannover, Hanover, Germany

**Keywords:** anxiolytic, imepitoin, *in vitro*, *in vivo*, dog, mouse, rat

## Abstract

Imepitoin is a low affinity partial agonist for the benzodiazepine binding site of γ-aminobutyric acid (GABA_A_) receptors, and is currently used as an antiepileptic in dogs. Here we tested imepitoin for anxiolytic properties. In an *in vitro* model, imepitoin was capable of preventing the effect of corticotrophin releasing factor (CRF) on locus coeruleus neurons without suppressing the basal activity of these cells, an activity which is suggestive for an anti-stress effect of imepitoin. In addition, we applied a battery of standard rodent preclinical tests for anxiety behavior including elevated plus mazes in mice and rats, light-dark-box in mice and rats, social interaction test in rats, or the Vogel conflict test in rats. In all models, the observed profile of imepitoin appeared similar to benzodiazepines and typical for anxiolytic drugs. We also observed anxiolytic activity in dogs in a provoked open field sound-induced fear model, where reactions to noises were elicited by a sound recording of thunderstorms. Imepitoin caused an increase in locomotion measured in distance traveled and an ameliorating effect on cortisol levels in response to thunderstorm noises. For comparison, dexmedetomidine caused a decrease in locomotion and had no effect on cortisol. In all animal models the doses needed for an anxiolytic effect were not associated with sedation. In rodents, there was at least a factor of 10 between anxiolytic doses and doses with mild signs of sedation. In summary, imepitoin showed similar anxiolytic activities as benzodiazepines but without producing the known adverse reactions of benzodiazepines such as sedation.

## Introduction

Physiologically, anxiety is an important protective mechanism to increase vigilance, which allows an individual to react to a perceived present or anticipated threat with an appropriate behavior (e.g., flight). When occurring at inappropriate times or to an excessive degree, anxiety can be described as pathological both in humans and in animals (Stein and Steckler, 2010). Despite the high prevalence of these conditions current treatment options are limited (Griebel and Holmes, 2013).

Since full agonist benzodiazepines are known to be potent anxiolytics, it was hypothesized that partial agonists could also potentially retain anxiolytic activity, but without producing the known adverse reactions of benzodiazepines such as sedation, muscle relaxation, and, upon repeated administration, development of tolerance. (Skolnick, 2012).

Imepitoin is a centrally acting drug and a low affinity partial agonist for the BZD binding site of γ-aminobutyric acid (GABA_A_) receptors (Rundfeldt and Loscher, 2014). The compound potentiates the amplitude of GABA evoked chloride (Cl-) currents by acting at the BZD recognition site of the GABA_A_ receptor, creating a higher affinity between GABA and its receptor sites. Imepitoin has a lower affinity for the BZD binding site of GABA_A_ receptors than BZD receptor agonists such as diazepam (DZP). Additionally it exhibits a weak calcium (Ca^2±^) channel blocking effect. Currently imepitoin is in veterinary use for the treatment of idiopathic epilepsy in dogs (Rundfeldt and Loscher, 2014).

Anxiolytic effects in the GABA-ergic pathway are mediated by α2- and α5 and possibly a3- subunit containing GABA_A_-receptors (Löw et al., 2000; Whiting, 2006). Previous work demonstrated that imepitoin is active on these receptor subtypes (Sigel et al., 1998).

Here we report results from preclinical studies examining anxiolytic effects of imepitoin. Based on imepitoin’s mode of action, we examine whether imepitoin had similar effects as benzodiazepines in commonly used models.

## Materials and Methods

### Locus Coeruleus (LC) Neuronal Cell Culture

Single locus coeruleus (LC) neurons in 200 μm thick brainstem slices were visualized with infrared videomicroscopy and patch-clamped in the whole-cell configuration as described elsewhere (Liss et al., 1999). These cells exhibit a spontaneous pace maker activity which can be reversibly potentiated with corticotrophin releasing factor (CRF). Drugs were directly applied onto the brain slices in concentrations of 0.1, 1, and 10 μM.

### Animals

All experiments were carried out in accordance with the principles of the Basel Declaration and according to applicable legislation [i.e., for example European directive on the protection of animals used for scientific purposes (2010/63/EU)]. If applicable, they were approved by the relevant authorities or Institutional Animal Care and Use Committees (IACUC).

Wistar rats (Crl WI BR, 270–330 g), CD rats (Crl 240–325 g) or Crl:NMRI BR mice (25–35 g) were used in the rodent experiments. Standard food (e.g., SNIFF Spezialdiäten GmbH, Soest, Germany) and water were provided *ad libitum*, and animals were housed in groups under a 12 h light and dark cycle.

Beagle dogs (Vivocore Inc., Fergus, ON, Canada) were group housed in pens of four with a hide box, a raised resting platform, various toys and background music (<85 decibels). A standard commercial diet once a day and water *ad libitum* was offered, and 12 h light and dark cycle was maintained.

The experimental setups were cleaned after each test (e.g., wiped with alcohol).

### Drug Preparation and Administration

For intraperitoneal and oral administration in rodents, all test substances were suspended in 5-hydroxyethylcellulose (0.5% in water), with plain 5-hydroxyethylcellulose (0.5% in water) solution serving as placebo. Presumably non-sedative doses for benzodiazepines were chosen, in consideration of the low oral bioavailability of benzodiazepines in rodents compared to humans and other species. In the dog study, tablets identical to the commercial presentation (Pexion^TM^; Boehringer Ingelheim Vetmedica GmbH, Ingelheim, Germany) were used, and baseline observations under no treatment were used as control. Administration of a single oral dose (20 mg/kg imepitoin) to dogs was performed 138 min before noise exposure, i.e., to achieve maximum plasma concentrations during the noise exposure (Rundfeldt et al., 2014). Dexmedetomidine as oromucosal gel (Sileo^TM^; Orion Pharma, Espoo, Finland) was administered 60 min before testing at a dose of 125 μg/m^2^ according to the manufacturer’s instructions.

### Elevated Maze in Rodents

For rats, the maze consisted of three open (35 × 10 cm) and three closed (35 × 10 × 15 cm) arms extending from a central platform. In mice, the maze consisted of two open (17.5 × 7.8 cm) and two closed (19 × 8 × 15 cm) arms extending from a central platform, placed 80 cm off floor (Walf and Frye, 2007).

In both cases, adaptation to the test room was ensured by 24 h housing in the test room and experiments were conducted under controlled light conditions (12 h on: 12 h off light dark cycle; lights on at 07.00 h, with normal ambient light). The movements of the animals were recorded for 5 min with a video camera for subsequent analysis. The number of entries in the open arms, the number of entries in the closed arms and the time spent in the open arms were measured (Walf and Frye, 2007).

Doses of imepitoin (1–30 mg/kg i.p.), alongside the benzodiazepines diazepam (0.25–2.0 mg/kg), triazolam (0.003–1.0 mg/kg), and alprazolam (0.1–1.0 mg/kg), administered 30 min before test, and pentetrazol (10.0 and 20.0 mg/kg) administered 5 min before test, were given to ten rats per group. In mice (*n* = 10 per group) i.p. doses of imepitoin (doses from 3.13 to 200 mg/kg), were compared to diazepam (0.2–12.5 mg/kg) and placebo administered 30 min before the test and p.o. administration of imepitoin 30 mg/kg 2 h before testing.

### Light-Dark-Box in Rodents

A two compartment box (each compartment 22 × 22 × 22 cm) was used in this test, with one light and open and the other dark and closed. Both compartments were connected with an open door. Animals were placed in the light part and observed for 5 min. Latency to first cross in the dark part, total time spent in light part and the number of crosses from one part into the other was measured (Bourin and Hascoet, 2003). Along with a placebo group, imepitoin was tested at different doses between 1 and 30 mg/kg and diazepam at doses between 1 and 10 mg/kg and placebo (*n* = 10 per group).

### Social Interaction Test in Rats

A pair of rats that were unfamiliar to each other was placed into an arena, one animal at each end (File and Hyde, 1978). Both rats were treated with the same test substance and dose. The arena was a circular open field apparatus made of white plastic, approximately 1 m in diameter enclosed by 25 cm high walls. It was covered by a muslin cloth and illuminated with ambient light. Behavior was scored at 15 s intervals over a 10 min period. The score values were 0 (No interaction), 1 (Passive interaction, e.g., lying in contact), 2 (one-way investigative interaction, e.g., 1 rat following or sniffing the other), 3 (one-way active interaction, e.g., 1 rat grooming the other), 4 (two-way investigative interaction, e.g., both rats mutually rat following or sniffing the other), or 5 (two-way active interaction, e.g., both rats grooming the other, playing together, etc.). The test was performed in five groups with nine pairs each with imepitoin (doses 3, 10, and 30 mg/kg), diazepam (5 mg/kg), and placebo.

### Conflict-Based Tests in Rodents

The Geller conflict test was performed as described elsewhere (Geller and Seifter, 1960). Rats were trained in a standard Skinner box (23 × 21 × 18 cm; MED Associates) that lever-pressing results in a food reward (food pellets 45 mg). The lever was located on the right of the food pellet dispenser, and above a white and a red signal light were installed. For further training and test, periods of responding by lever pressing to get food pellets on a variable interval schedule (mean value 15 s) indicated by a white light are interrupted by periods where, in the presence of a red signal light, food can be obtained more frequently (mean value 10 s) but accompanied by mild footshocks (0.4 mA, 0.5 s). Each lever pressing under white light (i.e., without electric shock) was defined as unpunished response, while those under red light were punished responses. The number of unpunished and punished responses was measured. Groups of eight rats received either imepitoin (3–30 mg/kg), diazepam (16 mg/kg), chlordiazepoxid (16 mg/kg), or placebo.

Briefly, the Vogel conflict test (Millan and Brocco, 2003) consisted of a Plexiglas conflict test box (30 × 23 × 19 cm). A water bottle was placed outside the box, with a stainless steel spout extending into the box about 2 cm above floor level. After an unpunished training session of 10 min, the second session lasted 3 min and was performed on the next day. In that session drinking was punished for 2-s periods, followed by 3 s unpunished periods. The number of electric shocks (0.5 mA) received within the 3 min test period was recorded. Groups of 12 rats received either imepitoin (1–50 mg/kg), diazepam (0.3–30 mg/kg), or placebo.

The four-plate apparatus used in mice is a rectangular chamber (23 × 18 × 30 cm) whose floor is divided into four equally sized rectangular copper plates, as described elsewhere (Jones et al., 1994). The test was divided into two parts, 20 s of free exploration, followed by a second phase in which each crossing between the plates was punished with a mild (1.0 mA) and brief (0.1 s) electric shock to the feet administered by the experimenter using a hand held button. The measure was the number of shocks received in 1 min period. Groups of 10 mice received either imepitoin (3.13–200 mg/kg), diazepam (0.2–12.5 mg/kg), or placebo.

### Noise-Induced Model of Fear and Anxiety in Dogs

For this test, subjects were placed in an open field arena, which is a room 2.45 × 2.75 m for 9 min (Araujo et al., 2013). It was hypothesized that if a drug has anxiolytic properties then it should result in a shift from fearful flight to behavioral inhibition or non-anxious “rumination” (McNaughton and Gray, 2000) and an increase in exploratory behavior. This would be manifest as an increase in behavioral pauses, but an increase in behavioral activity (since exploratory behavior is more elaborate than flight behavior), without an increase in cortisol in cortisol reactive subjects (raised cortisol being indicative of increased arousal associated with fear response in this context). Animal behavior was recorded via video and distance traveled, as well as frequency and duration of inactivity (defined as the animal sitting or lying down, or not exhibiting any overt behavior) were measured using Ethovision XT (Noldus Information Technology, Netherlands). Blood samples for cortisol measurements were collected 1 h before and 5 min after the test.

For training, each subject was allowed to freely explore the room over a 9-min period during which no stimuli were provided. For baseline and test measurements, a thunder track played over a stereo system with a decibel range of 61–85 db for 3 min of the 9 min period (minutes 4–6).

Beagle dogs (both gender, age 1–6 years) were first grouped based on their cortisol response into cortisol responders (at least a 9% increase in blood cortisol from the baseline) and non-responders, and secondly on their activity responses as hyperactive responders (≥10% increase in activity ratio between minutes 1–3 and minutes 4–6 from training to baseline thunderstorm) and hypoactive responders (<10% increase). In two independent experiments, 24 dogs received imepitoin and 25 dogs dexmedetomidine. Baseline values from the respective experiment values served as control. Drugs were administered as a single dose before the test (imepitoin 135 min, dexmedetomidine 60 min before thunder test).

### Statistics

Treatment allocation was performed randomly and remained completely blinded for the examiners. Data are presented descriptively as mean and SD, or for ordinal values as median. Hypothesis tests were always performed against placebo control or baseline, using appropriate 2-sided statistical tests for significant differences (*p* < 0.05; mainly Mann–Whitney *U* or *t*-Test for two groups, Dunnett’s procedure for more than two groups).

For logistical reasons, most of the animal tests were set up as a series of experiments. A control group was included in each single experiment, and wherever possible, all doses of a test product were tested in the same experiment.

## Results

### Effect on Basal and CRF-Induced Activity in the Locus Coeruleus (LC) Neurons

**FIGURE 1:**
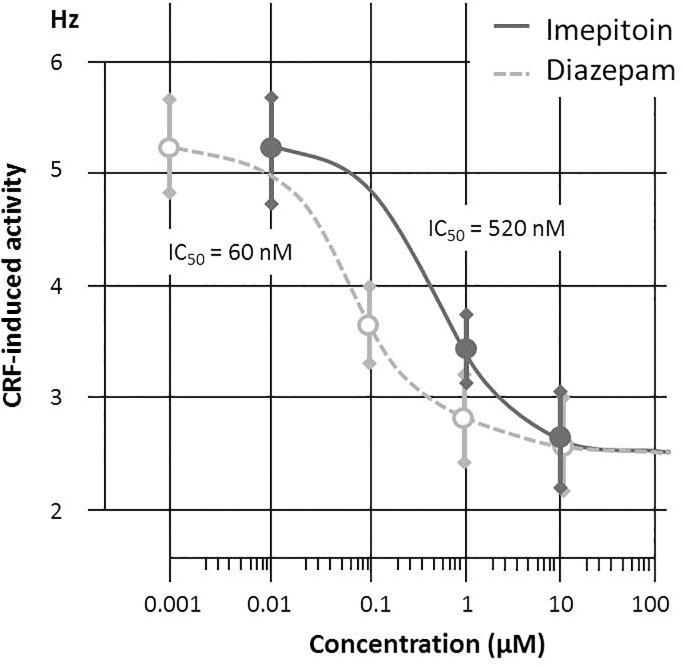
Corticotrophin Releasing Factor (CRF, 200 nM) induced discharge of mouse locus coeruleus neurons are reduced by both Diazepam (doses 0.1; 1 and 10 μM) and imepitoin (doses 1 and 10 μM) with a half maximal inhibition in the range of 60 and 520 nM, respectively (*n* = 3 per concentration).

The *in vitro* study indicated that imepitoin (at doses of 1 and 10 μM) acts to decrease neuronal activity induced by CRF but not the basal (spontaneous pacemaker) activity in mouse LC neurons. The half-maximal inhibition could be determined to 60 nM for diazepam and 520 nM for imepitoin, respectively (Figure 1).

### Elevated Maze in Rodents

**FIGURE 2:**
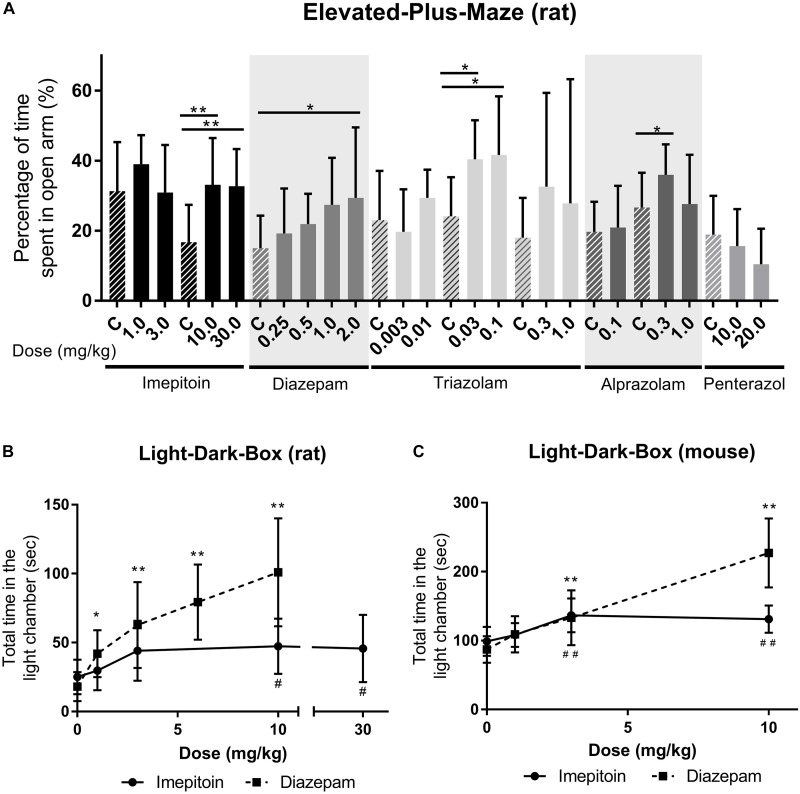
**(A)** Imepitoin at doses of 10.0 and 30.0 mg/kg shows significantly increased percentage of time spent in the open arm of the elevated maze in rats compared to placebo control (marked as “C”). Similar effects were observed with different benzodiazepines, and the anxiogenic substance penterazol led to a decreased percentage. (Series of 9 experiments, *n* = 10 per group) **(B)** In rats, the total amount of time spent in the light part of the light-dark-box was significantly increased in doses above 1.0 mg/kg imepitoin compared to control (0 mg/kg) (Series of three experiments, *n* = 10 per group) **(C)** A similar effect was observed in the light-dark-box in mice (Series of four experiments, *n* = 10 per group). For all displays: mean ± SD; comparison against respective control using ANOVA with Dunnett’s *post hoc*. ^∗^ or # *p* < 0.05; ^∗∗^ or ## *p* < 0.01; and the symbol “#” indicates imepitoin and “^∗^” indicates diazepam.

The influence of imepitoin on anxiety related behavior of rats (*n* = 10 per group) was investigated in an elevated maze. At doses of 10 and 30 mg/kg i.p. (administered 30 min before test) the percentage of entries in the open arms and the percentage of time spent in the open arms were significantly increased compared to placebo. The doses 1 and 3 mg/kg i.p. did not show significant differences to placebo (Figure 2A, Table 1, and Supplementary Table 1).

**Table 1:** Results of model studies in rats and mice.

*Treatment*	*Dose*	*Measure*	*Statistical result*	*Treatment*	*Dose*	*Measure*	*Statistical result*
**Elevated Maze in Rats**. Parameter: % time spent in open arms. Statistical Test: Dunnett’s procedure. *N* = 10 per group.							

Imepitoin	C	31.3 ± 14.0	*F*(2,27) = 1.379	Triazolam	C	24.1 ± 11.19	*F*(2,27) = 5.392^∗^
	1.0	39.0 ± 8.25	(ns)		0.03	40.4 ± 11.16	*p* < 0.05
	3.0	30.9 ± 13.57	(ns)		0.1	41.6 ± 16.79	*p* < 0.05
	C	16.7 ± 10.69	*F*(2,27) = 6.493^∗^		C	18.0 ± 11.35	*F*(2,27) = 0.790
	10.0	33.1 ± 13.34	*p* < 0.01		0.3	32.6 ± 26.78	(ns)
	30.0	32.7 ± 10.59	*p* < 0.01		1.0	27.8 ± 35.48	(ns)
Diazepam	C	15.0 ± 9.3	*F*(4,45) = 2,633^∗^	Alprazolam	C	19.7 ± 8.57	[see footnote]
	0.25	19.2 ± 12.84	ns		0.1	20.9 ± 11.89	ns
	0.5	21.9 ± 8.66	ns		C	26.6 ± 9.96	*F*(2,27) = 3.884^∗^
	1.0	27.4 ± 13.41	ns		0.3	35.97 ± 8.7	*p* < 0.05
	2.0	29.4 ± 20.08	*p* < 0.05		1.0	27.6 ± 14.07	ns
Triazolam	C	23.0 ± 14.07	*F*(2,27) = 1.783	Penterazol	C	18.9 ± 11.07	*F*(2,27) = 1.631
	0.003	19.7 ± 12.14	(ns)		10.0	15.6 ± 10.56	(ns)
	0.01	29.4 ± 8.00	(ns)		20.0	10.4 ± 10.18	(ns)

**Elevated Maze in Mice**. Parameter: Entries into open arms. Statistical Test: Dunnett’s procedure. *N* = 10 per group.							

Imepitoin	C	9,8 ± 3,79	*F*(7,72) = 2.52^∗^	Diazepam	C	9 ± 2,53	*F*(7,72) = 3.13^∗^
	3.1	16 ± 5,69	*p* < 0.05		0.2	13 ± 5,38	ns
	6.25	10,8 ± 3,16	ns		0.39	16,7 ± 6,96	ns
	12.5	11 ± 4,11	ns		0.78	15,5 ± 6,96	ns
	25	12,4 ± 4,74	ns		1.56	23,1 ± 8,85	*p* < 0.01
	50	7,8 ± 4,43	ns		3.13	14,6 ± 11,70	ns
	100	9,1 ± 8,85	ns		6.25	16,3 ± 12,65	ns
	200	5,8 ± 4,74	ns		12.5	9,3 ± 8,22	ns

**Light Dark in rats**. Parameter: Total time in light chamber. Statistical Test: Dunnett’s procedure. *N* = 10 per group.							

Imepitoin	C	25 ± 12,6	*F*(4,45) = 2.866^∗^	Diazepam	C	18 ± 10,5	*F*(4,45) = 14,18^∗^
	1	29,7 ± 14,29	ns		1	41,9 ± 17,08	*p* < 0.05
	3	44,1 ± 21,82	ns		3	62,7 ± 31,15	*p* < 0.01
	10	47,3 ± 20,02	*p* < 0.05		6	79,3 ± 27,26	*p* < 0.001
	30	45,7 ± 24,35	*p* < 0.05		10	100,9 ± 39,21	*p* < 0.001

**Light Dark in mice**. Parameter: Total time in light chamber. Statistical Test: Dunnett’s procedure. *N* = 10 per group.							

Imepitoin	C	98,8 ± 21,19	*F*(3,36) = 7,427^∗^	Diazepam	C	87 ± 19,29	*F*(3,36) = 29,6^∗^
	1	108 ± 17,39	ns		1	109 ± 26,25	ns
	3	136,5 ± 24,67	*p* < 0.001		3	133 ± 39,84	*p* < 0.05
	10	131 ± 19,92	*p* < 0.01		10	227,2 ± 49,96	*p* < 0.0001

**Social Interaction in rats**. Parameter: score points. Statistical Test: Kruskal–Wallis with Dunn’s *post hoc*. *N* = 9 pairs per group.							

Imepitoin	C	62.89 + 10,76	*H* = 16.11^∗^	Imepitoin	30	60.67 + 8.68	ns
	3	58 + 13,26	ns	Diazepam	5	70.78 + 8.18	ns
	10	78.56 + 13.19	*p* < 0.05				

**Vogel Conflict Test in rats**. Parameter: number of shocks received. Statistical Test: Dunnett’s procedure. *N* = 12 per group.							

Imepitoin	C	51,4 ± 26,67	*F*(5,66) = 9,147^∗^	Diazepam	C	62,3 ± 22,35	*F*(3,44) = 6,08^∗^
	1	61,3 ± 30,14	ns		0.3	70,9 ± 23,73	ns
	3	83,2 ± 24,94	ns		1	92,1 ± 11,15	*p* < 0.05
	10	80 ± 31,87	ns		3	104 ± 41,22	*p* < 0.01
	30	126 ± 54,39	*p* < 0.0001				
	50	135 ± 53,69	*p* < 0.0001				

**Geller Conflict test in rats**. Parameter: Number of punished responses. Statistical Test: Dunnett’s procedure. *N* = 8 per group.							

Chlordiazepoxide	C	1,6 ± 2,26	*t* = 4,078 *df* = 14	Imepitoin	10	2,6 ± 3,39	(ns)
	16	10 ± 5,37	*p* < 0.01		30	2,4 ± 2,55	(ns)
Imepitoin	C	2,9 ± 5,37	*F*(4,35) = 0,6268	Diazepam	16	5,4 ± 5,37	(ns)
	3	4,6 ± 6,22	(ns)				

**Four Plate Test in mice**. Parameter: punished crossings. Statistical Test: Dunnett’s procedure. *N* = 10 per group.							

Imepitoin	C	14.7 ± 4.74	*F*(7,72) = 0.99	Diazepam	C	14.8 ± 7.91	*F*(7,72) = 3.4^∗^
	3.13	16.1 ± 6.01	(ns)		0.2	23.1 ± 14.23	ns
	6.25	17.1 ± 8.22	(ns)		0.39	27.1 ± 12.65	*p* < 0.05
	12.5	14.9 ± 7.59	(ns)		0.78	25.7 ± 8.22	ns
	25	14.0 ± 7.27	(ns)		1.56	27.7 ± 7.91	*p* < 0.05
	50	16.6 ± 13.28	(ns)		3.13	28.2 ± 15.50	*p* < 0.05
	100	13.9 ± 8.22	(ns)		6.25	14.7 ± 7.59	ns
	200	9.2 ± 7.91	(ns)		12.5	16.4 ± 9.17	ns


Dosage “C” indicates placebo control. All measures presented as mean ± SD. For ANOVA procedures *F*(dFn,dFd) is reported with ^∗^ indicating significance (i.e., *p* < 0.05). All *post hoc* tests were performed for differences compared to control. ns, non-significant differences. For Alprazolam 0.1 mg/kg: As this experiment was only a two group set-up, no ANOVA could be performed. An unpaired *t*-test revealed no significant differences (*t* = 0.2589; *df* = 18).

For comparison the benzodiazepines diazepam (0.25, 0.5, 1.0, and 2.0 mg/kg), triazolam (0.003, 0.01, 0.03, 0.1, 0.3, and 1.0 mg/kg), and alprazolam (0.1, 0.3, and 1.0 mg/kg) were tested (all i.p. administered 30 min before test). Diazepam significantly increased the percentage of time spent in the open arms (Table 1) at doses of 1 and 2 mg/kg compared to placebo. For triazolam the percentage of entries was significantly increased for doses of 0.03 mg/kg and above (Table 1), and the time spent in the open arm was significantly increased for 0.03 and 0.1 mg/kg, while in higher doses sedative effects were observed. Alprazolam significantly increased the percentage of time spent in the open arms at 0.3 mg/kg (Table 1), while there were sedative effects at the high dose.

In contrast the anxiogenic compound pentetrazol (10.0/20.0 mg/kg administered 5 min before test) was tested. The higher dose decreased the percentage of time spent in the open arms compared to placebo (Table 1).

In mice (*n* = 10 per group) the elevated maze model was used with various i.p. doses of imepitoin (3.13, 6.25, 12.5, 25, 50, 100, and 200 mg/kg), diazepam (0.2, 0.39, 0.78, 1.56, 3.13, 6.25, and 12.5 mg/kg), and placebo administered 30 min before the test. After low dose i.p. administration of imepitoin (3.13 mg/kg) we observed an increased number of entries into the open arm (Table 1). All other variables and doses did not reveal significant treatment effects (Supplementary Table 1). In the highest administered doses of imepitoin (100 and 200 mg/kg) slight locomotor sedation was observed in mice. Diazepam increased the number of entries into the open arm with a minimal effective dose of 0.39 mg/kg (Table 1). At high doses of diazepam (≥6.25 mg/kg) there were indications of sedative effects. At doses of 3.13 mg/kg and 6.25 mg/kg diazepam, a small number of animals fell off the maze (data not shown).

In mice, after p.o. administration of 30 mg/kg imepitoin 2 h prior to the test no effect was observed compared to placebo.

### Light-Dark-Box in Rodents

The effect on the latency to the first cross to the dark part of the box, the total amount of time spent in the light part and the number of crosses were assessed.

In rats (*n* = 10 per group) imepitoin was tested in a dose range of 1, 3, 10, and 30 mg/kg po compared to placebo (administered 2 h before test). The latency to first cross and total time in the light chamber were significantly increased at doses higher than 1 mg/kg imepitoin (Table 1), however, no clear dose dependence was observed. The number of movements between the compartments was not significantly increased. No visible side effects occurred within the dose range tested. For comparison, diazepam (1, 3, 6, and 10 mg/kg oral, 30 min before test) showed a strong anti-anxiety response in a dose-dependent manner (Figure 2B; parameters with significant effects: latency to the first cross, total time in the light chamber, number of crosses).

In mice (*n* = 10 per group), imepitoin at i.p. doses of 3 and 10 mg/kg (administered 30 min before test) significantly prolonged the phases of staying in the light part of the two chamber box in mice compared to placebo. The dose 10 mg/kg additionally prolonged the latency before the mice entered for first time the dark compartment. The dose 1 mg/kg showed no changes compared to placebo. Diazepam (1, 3, and 10 mg/kg) exerted its anxiolytic like activity in a dose dependent manner (Figure 2C and Table 1).

### Social Interaction Test in Rats

**FIGURE 3:**
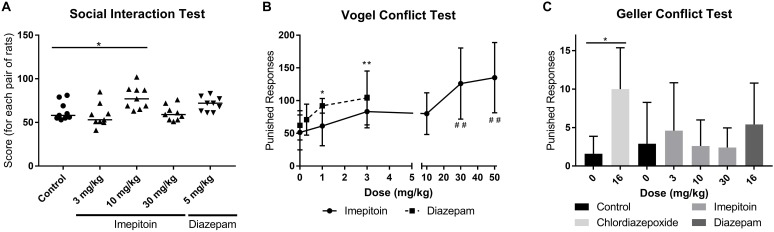
**(A)** Treatment with 10 mg/kg imepitoin significantly increased the interaction between pairs of rats (*n* = 9 pairs per group). **(B)** An increase in the number of punished responses in the Vogel conflict test was observed in doses above 10 mg/kg imepitoin or above 1 mg/kg diazepam (Series of three experiments, *n* = 12 per group). **(C)** No relevant effect was observed in the Geller conflict test neither for imepitoin nor for diazepam (*n* = 8 in cross over design). For all tests: mean ± SD. Comparison against respective control using ANOVA with Dunnett’s *post hoc*.^∗^ or # *p* < 0.05; ^∗∗^ or ## *p* < 0.01 In display **B**, the symbol “#” indicates imepitoin and “^∗^” indicates diazepam.

The effect on social interaction was assessed in pairs of unfamiliar rats (5 groups with 9 pairs each) in unfamiliar surroundings under high illumination. Imepitoin administered by oral gavage once daily for four consecutive days, significantly increased social interaction at 10 mg/kg compared to placebo (Table 1) but had no significant effect on social interaction at either 3 or 30 mg/kg. Signs of sedation were noted in some rats treated with 30 mg/kg which may have interfered with social interaction. The positive control, diazepam 5 mg/kg similarly administered by oral gavage likewise increased social interaction (Figure 3A and Table 1).

### Conflict-Based Tests in Rodents

The anxiolytic effect of oral imepitoin (1, 3, 10, 30, and 50 mg/kg), diazepam (0.3, 1, and 3 mg/kg), or placebo in rats was also investigated using the Vogel conflict test. Imepitoin exerted a dose dependent increase in the number of shocks received, which was significantly increased at doses of 30 and 50 mg/kg compared to placebo (Figure 3B and Table 1). The number of shocks was also increased for diazepam in a dose-dependent manner being significant at doses of 1 and 3 mg/kg (Table 1).

Imepitoin (3, 10, and 30 mg/kg as oral suspension, administered 2 h before the test) was also tested in the Geller conflict test in rats (*n* = 8 in cross over design), with chlordiazepoxide (16 mg/kg p.o. 1 h prior to test), diazepam (16 mg/kg p.o., 2 h prior to test) and placebo for comparison.

After administration of 3 mg/kg imepitoin or 16 mg/kg diazepam tended to increase the number of punished responses, however, the difference did not reach statistical significance compared to placebo. No effects were observed for 10 and 30 mg/kg imepitoin. In this study, only chlordiazepoxide after administration of a dose exerting sedative effects (16 mg/kg) significantly increased the number of punished responses and the number of shocks received (Figure 3C and Table 1). Imepitoin did not affect the number of unpunished responses at any dose tested, i.e., the substance did not show sedative effects.

In mice, the four plate test was used with intraperitoneal administration of imepitoin (3.13, 6.25, 12.5, 25, 50, 100, and 200 mg/kg), diazepam (0.2, 0.39, 0.78, 1.56, 3.13, 6.25, and 12.5 mg/kg), and placebo administered 30 min before the test. No effect on punished as well as on un-punished behavior was shown for imepitoin (3.13–200 mg/kg i.p), whereas diazepam (0.39–3.13 mg/kg i.p) significantly increased crossings of punished plates compared to placebo (Table 1). In higher doses, diazepam elicited sedative effects.

### Noise-Induced Model of Fear and Anxiety in Dogs

**FIGURE 4:**
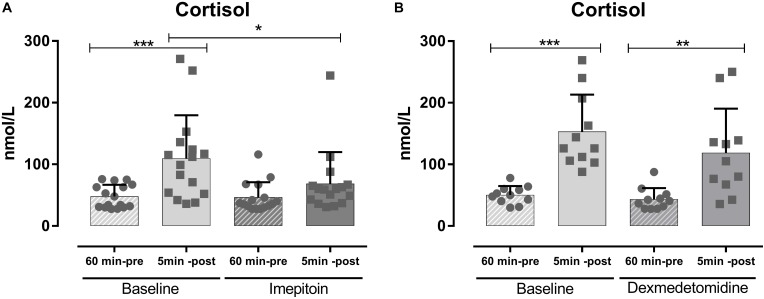
In an open field noise-induced fear model (i.e., exposure to thunderstorm noises) serum cortisol values were measured 60 min before and 5 min after exposure to noise. **(A)** In a first experiment (*n* = 12), single dose imepitoin treatment (20 mg/kg) was compared to baseline. **(B)** The same setup was independently repeated (*n* = 25) with a single dose of dexmedetomidine. Mean ± SD; ^∗^*p* < 0.05; ^∗∗^*p* < 0.01; and ^∗∗∗^*p* < 0.001 in ANOVA with Sidak’s *post hoc*.

**FIGURE 5:**
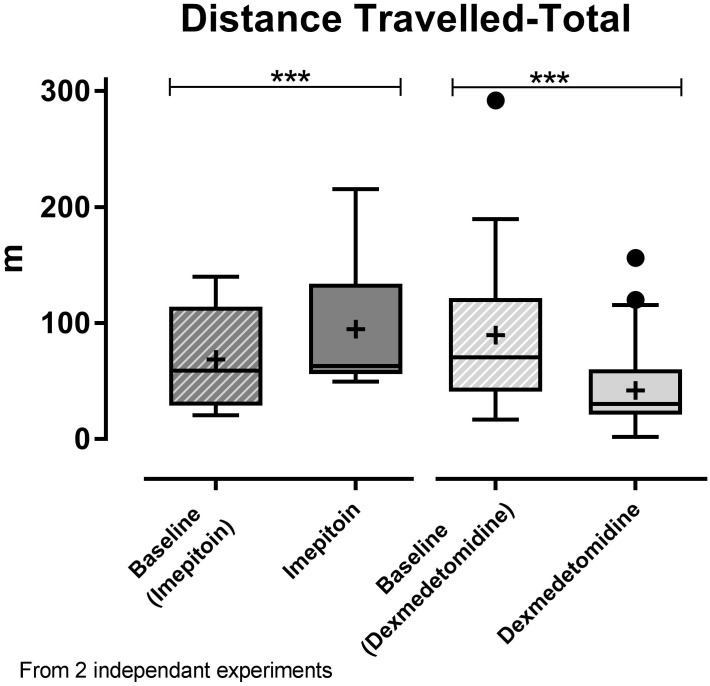
In the two independent noise-induced fear model experiments, a single dose of imepitoin (20 mg/kg; *n* = 12; left) elicited an increase in distance traveled compared to baseline, while a single dose of dexmedetomidine (125 μg/m^2^; *n* = 25; right) resulted in a decrease compared to baseline. Boxplot with whiskers according to Tukey’s procedure and “+”indicating the mean; ^∗∗∗^*p* < 0.001 in paired *t*-test.

Dogs (*n* = 24) were tested in an open field sound-induced fear model with exposure to artificial stimuli (thunderstorm sound) as eliciting context and treated with a single dose of imepitoin (20 mg/kg p.o.) 138 min before thunder exposure. Overall, imepitoin significantly lowered the cortisol levels in 13 out of 16 dogs (81%) that were declared as cortisol responders at the beginning of the study (Figure 4A). Both during the complete testing period as well as during the thunder interval (minutes 4–6), total distance traveled was significantly higher during the treatment thunder test compared to the baseline thunder test (Figure 5 and Table 2). In addition, total inactivity frequency was significantly higher at treatment than baseline.

**Table 2:** Results of model studies in dogs.

*Parameter*	*Timepoint*	*Measurement*	*Statistical result*	*Measurement*	*Statistical result*
			
		Imepitoin		Dexmedetomidine	
Cortisol	Baseline 60 min pretest	47.9 ± 19.02 (16)	*F*(3,28) = 3,248^∗^	50.49 ± 14.4 (11)	*F*(3,40) = 13,39^∗^
	Baseline 5 min post test	109.7 ± 69.78 (16)	*p* < 0.001 vs. Baseline 60 min	153.1 ± 60.16 (11)	*p* < 0.001 vs. Baseline 60 min
	Treatment 60 min pretest	46.49 ± 24.36 (16)		43.57 ± 17.98 (11)	
	Treatment 5 min posttest	68.54 ± 51.32 (16)	*p* < 0.05 vs. Baseline 5 min	118.6 ± 71.9 (11)	*p* < 0.01 vs. Treatment 60 min
Distance traveled total	Baseline Total time	89.61 ± 50.4	*t* = 3,939 *df* = 23	89.69 ± 64.55	*t* = 6,045 *df* = 24
	Treatment Total time	123.4 ± 69.26	*p* < 0.001	42.12 ± 38.73	*p* < 0.001
Inactivity frequency	Baseline	19.08 ± 8.607	*t* = 2.419 *df* = 23	13.68 ± 10.75	*t* = 4,156 *df* = 24
	Treatment	24.04 ± 11.26	*p* < 0.05	9.12 ± 7.677	*p* < 0.001


*All measures presented as mean ± SD. For ANOVA procedures *F*(dFn,dFd) is reported with ^∗^ indicating significance (i.e., *p* < 0.05). ns, non-significant differences. For two-group comparisons, paired t-test between baseline and treatment was used.*

Separating the dogs into hypoactive (i.e., those who reacted at baseline with less activity in response to thunder noise) and hyperactive (i.e., those who reacted at baseline with more activity in response to thunder noise), in both groups (*n* = 12 each) an increase in distance traveled and inactivity frequency was observed. However, the effect was larger for the hypoactive dogs and was significant in this subgroup only (distance traveled: *p* < 0.001 in paired *t*-test; inactivity frequency: *p* < 0.05).

In contrast, in a separate experiment dogs (*n* = 25) treated with dexmedetomidine applied as oromucosal gel showed a significantly decreased total distance traveled (Figure 5 and Table 2). Dexmedetomidine showed no relevant effect on cortisol levels in dogs that were declared as cortisol responders at the beginning of the study (Figure 4B and Table 2).

## Discussion

Imepitoin was tested in a battery of standard preclinical tests for anxiety behavior. The observed profile appears typical for anxiolytic drugs (Andrews et al., 2014). Based upon the performance of imepitoin in these standard tests designed to evaluate potential anxiolytic activity, it can be concluded that imepitoin has anxiolytic properties similar to benzodiazepines.

In an *in vitro* model, imepitoin was capable of preventing the effect of CRF on LC neurons without suppressing the basal activity of these cells, an activity which is suggestive for an anti-stress effect of imepitoin (Valentino et al., 1993).

Ethological test paradigms rely on stimulated forms of anxiety within the normal range. The elevated-plus-maze for example is based on small rodent’s avoidance of open, bright areas due to the higher risk of predator attacks (Hogg, 1996; Rodgers et al., 1997). Especially the elevated-plus maze appears to be sensitive to positive GABA modulating agents, while other substance classes like for example α_2_-agonists (e.g., dexmedetomidine) showed no effect in this test (Salonen et al., 1992). The social interaction test reflects the uncertainty in meeting unknown individuals in unfamiliar environments, and this ethologically natural paradigm can be used to examine social avoidance (File and Seth, 2003). In all of the tests applied, imepitoin showed comparable responses to diazepam, however, at higher doses. This observation supports the notion that imepitoin acts as a partial agonist.

In addition, conflict-based tests were used, measuring anxiolytic-like activity as the maintenance of a behavioral response (for example, licking or bar pressing) despite the receipt of a mild electric shock. Anxiolytic action in these tests is generally defined as maintenance of responding in the presence of the punishing stimulus since this would override the inhibition. Interpretation of the results appears difficult under the premise that disinhibition alone is not *per se* an anxiolytic action, and analgesic effects of a test compound could produce similar effects simply by inhibiting pain reactions to the punishment (Andrews et al., 2014). In the Geller conflict test a large variability in response to certain benzodiazepines is described in the literature (Babbini et al., 1982). Regardless, conflict situations with two opposing impulses are a component of anxiety disorders, and conflict based tests are still widely applied to screen for anxiolytic drug candidates. Imepitoin showed only a significant effect in the Vogel conflict test, but not in the Geller conflict test or the four plate test. Apparently imepitoin has no strong disinhibition component and does not result in irrational risk taking.

We observed anxiolytic activity also in dogs in a provoked open field sound-induced fear model, where reactions to noises were elicited by a sound recording of thunderstorms. While tests like these are considered to have a good test–retest reliability, the generalizability to patients relating to changes in locomotion and exploratory behavior observed under anxiolytic treatment underlies the general limitations of a model (Wormald et al., 2016). We observed an increase in locomotion measured in distance traveled after imepitoin treatment. In addition, dogs paused their activity more often under treatment, which might indicate an interruption of their current behavior to assess their environment (McNaughton and Gray, 2000). After dexmedetomidine treatment, which was shown to have anxiolytic effects in dogs (Korpivaara et al., 2017), we observed a decrease in locomotion. This is not a surprising result as dexmedetomidine, and other α2-adrenoceptor agonists, are widely used as premedication to induce anesthesia (Beckman, 2013) and sedative effects were already described for transmucosal administration in dogs (Cohen and Bennett, 2015). For comparison, the effect of benzodiazepines in this model is associated with an increase in exploratory behavior, which is also measured as increase in total distance traveled (Wormald et al., 2016). Due to the highly controlled conditions, the changes in behavior in the imepitoin or dexmedetomidine groups compared to baseline are most plausibly explained by the treatment.

Cortisol as a stress hormone indicative of arousal plays a role in anxiety disorders. For example, dogs with noise phobia suffer from severe mental stress evidenced by an inability to relax and cortisol levels in these dogs increasing more than twofold during thunderstorms (Dreschel and Granger, 2005). In addition, dogs with noise phobia have higher cortisol levels in their hair (Siniscalchi et al., 2013). In our hands, we observed an ameliorating effect of imepitoin on cortisol levels in response to thunderstorm noises, but not for dexmedetomidine. Benzodiazepines were found to suppress cortisol levels (Manthey et al., 2010), and thus these findings further support the benzodiazepine-like anxiolytic effect of imepitoin.

A relevant limitation of the suite of studies presented here is the use of different dosages and administration routes across studies. While the different dosages were chosen based on the stage of the development program reported here, it complicates comparison between the models and allows no definitive conclusions on the optimal dose. Notably, the logistical need for separate independent experiments due to the large number of groups limits the comparison between treatments and between independent experiments to a qualitative assessment. In addition, the conclusion of an anxiolytic effect is based on the similar performance to benzodiazepines in standard models of anxiety, which needs validation in clinical trials.

In all animal models the imepitoin doses needed for an anxiolytic effect were not associated with sedation. In rodents, there was at least a factor of 10 between anxiolytic doses and doses with mild signs of sedation. In the dog model, treatment with imepitoin resulted in increased locomotion.

## Conclusion

In conclusion, imepitoin showed anxiolytic properties similar to benzodiazepines in various standard models for anxiety behavior but without producing the known adverse reactions of benzodiazepines such as sedation, without evidence of behavioral inhibition in the situations explored. Accordingly imepitoin appears to be a promising candidate for treating anxiety disorders, and first data in dogs with anxiety problems showed promising results for therapeutic use of imepitoin in canine anxiety disorders (McPeake and Mills, 2017).

## Availability of Data and Material

The raw data supporting the conclusions of this manuscript will be made available by the authors, without undue reservation, to any qualified researcher.

## Author Contributions

All authors had access to all the study information, raw data, and approved the manuscript. CR led the conduct and evaluation of rodent models. DM, OE, GL, and AM contributed to the conception, design of the dog anxiety model, and the interpretation of the results. AM and MB conducted and evaluated the dog open field studies. OE prepared the manuscript with support of all authors.

## Conflict of Interest Statement

This publication followed the GPP3 guidelines. OE is an employee of Boehringer Ingelheim Vetmedica GmbH, Germany, the marketing authorization holder of Pexion^®^, containing Imepitoin as active principle. CR and DM acted as consultants for Boehringer Ingelheim.The remaining authors declare that the research was conducted in the absence of any commercial or financial relationships that could be construed as a potential conflict of interest.

## Abbreviations

SD: standard deviation
