# Supplementary material for: Imepitoin Shows Benzodiazepine-Like Effects in Models of Anxiety
Source: Front Pharmacol. 2018 Nov 1;9:1225. doi: 10.3389/fphar.2018.01225 (PMC6230983; doi:10.3389/fphar.2018.01225)
Supplement: Supplementary file 1 [file Data_Sheet_1.PDF]

Supplementary Material

Imepitoin shows benzodiazepine-like effects in models of anxiety.

Odilo Engel[1]*, Alexandar Masic[2], Gary Landsberg[2], Melissa Brooks[2], Daniel Mills[3], Chris Rundfeldt[4,5]

Supplementary Table 1: Time spent in open arms in minutes for rats and mice. While imepitoin (at doses of 10 and 30 mg/kg) showed an effect on time spent in open arms in rats, this was not observed in mice.

Treatment	*Dose*	*Measure*		*Treatment*	*Dose*	*Measure*
**Elevated Maze in Rats**. Parameter: Time (in min) spent in open arms. N=10 per group						
Imepitoin	C	1.57 ± 0.70		Triazolam	C	1.21 ± 0.56
	1.0	1.95 ± 0.41			0.03	2.02 ± 0.55
	3.0	1.55 ± 0.68			0.1	2.08 ± 0.84
	C	0.84 ± 0.53			C	0.90 ± 0.57
	10.0	1.66 ± 0.67			0.3	1.63 ± 1.34
	30.0	1.64 ± 0.53			1.0	1.39 ± 1.77
Diazepam	C	0.75 ± 0.46		Alprazolam	C	0.99 ± 0.43
	0.25	0.96 ± 0.64			0.1	1.05 ± 0.59
	0.5	1.10 ± 0.43			C	1.33 ± 0.50
	1.0	1.37 ± 0.67			0.3	1.80 ± 0.43
	2.0	1.47 ± 1.00			1.0	1.38 ± 0.70
Triazolam	C	1.15 ± 0.70		Penterazol	C	0.95 ± 0.55
	0.003	0.99 ± 0.61			10.0	0.78 ± 0.53
	0.01	1.47 ± 0.40			20.0	0.52 ± 0.51
**Elevated Maze in Mice.** Parameter: Time (in min) spent in open arms. N = 10 per group						
Imepitoin	C	1.17 ± 0.45		Diazepam	C	1.09 ± 0.37
	3.1	1.71 ± 0.54			0.2	1.44 ± 0.40
	6.25	1.27 ± 0.39			0.39	1.49 ± 0.54
	12.5	1.39 ± 0.53			0.78	1.38 ± 0.49
	25	1.74 ± 0.75			1.56	2.20 ± 0.74
	50	1.29 ± 0.84			3.13	1.83 ± 1.40
	100	1.28 ± 1.13			6.25	2.62 ± 0.92
	200	1.25 ± 1.02			12.5	3.01 ± 0.67

Total observation time in maze: 5.00 min
